# The Mediating Role of Depressive Symptoms, Hopelessness, and Perceived Burden on the Association Between Pain Intensity and Late-Life Suicide in Rural China: A Case–Control Psychological Autopsy Study

**DOI:** 10.3389/fpsyt.2021.779178

**Published:** 2021-12-13

**Authors:** Jiali Wang, Jiahuan Xu, Zhenyu Ma, Cunxian Jia, Guojun Wang, Liang Zhou

**Affiliations:** ^1^Xiangya School of Public Health, Central South University, Changsha, China; ^2^Guangzhou Medical University, Guangzhou, China; ^3^School of Public Health, Guangxi Medical University, Nanning, China; ^4^School of Public Health, Shandong University, Jinan, China; ^5^Shenzhen Kangning Hospital, Shenzhen, China; ^6^The Affiliated Brain Hospital of Guangzhou Medical University (Guangzhou Huiai Hospital), Guangzhou, China

**Keywords:** suicide, older adults, depressive symptoms, hopelessness, perceived burden, pain intensity

## Abstract

**Background:** Few studies have investigated the roles of psychosocial factors such as depressive symptoms and hopelessness on the relationship between pain and suicide with inconsistent results. The study aimed to analyze the impact of pain intensity on suicide death and to estimate the degree to which depressive symptoms, hopelessness, and perceived burden may explain the association in Chinese rural elderly.

**Methods:** Using a 1:1 matched case–control design, we collected data from 242 elderly suicide cases and 242 living community controls by psychological autopsy method in rural China, including sociodemographic characteristics, pain intensity, depression, hopelessness, perceived burden, physical diseases, and social support. Conditional logistic regression was employed to assess the association between pain intensity and completed suicide. Mediation analysis using the KHB method was applied to explore the mediation effects from depressive symptoms, hopelessness, and perceived burden.

**Results:** The result of multivariable logistic regression showed that unemployment [odds ratio (OR) = 5.06, 95% confidence interval (CI): 1.76–14.49], higher levels of hopelessness (OR = 7.72, 95% CI: 3.49–17.10), depressive symptom (OR = 15.82, 95% CI: 4.53–55.25), and severe pain (OR = 3.46, 95% CI: 1.31–9.13) were significantly associated with elevated suicide risk in older adults in rural China. Depressive symptoms, hopelessness, and perceived burden significantly mediated 43.71% of the pain–suicide association (*p* = 0.020), with 17.39% due to depressive symptoms, 17.63% due to hopelessness, and 8.69% due to perceived burden.

**Conclusions:** Regular screening of pain, depressive symptoms, hopelessness, and perceived burden using simple but sensitive questions or scales for older adults with pain is vital for the prevention and early detection of suicide risk in Chinese rural areas. Moreover, the importance of pain management and psychological interventions targeted on depressive symptoms and hopelessness should be emphasized.

## Introduction

Though the suicide rate in China has declined rapidly, the elderly population increasingly contributes to the overall burden of suicide (1). The average annual suicide rate among old adults was estimated to be 6.5-fold higher than the rate of the population under 65, and the rate in rural older adults was two times higher than that in urban elderly (2). Suicide among older adults in rural China remains a critical public health and mental health problem. Identification of potential risk factors for this vulnerable population is of paramount significance to design targeted suicide preventions.

Pain is highly prevalent, costly, and frequently disabling in later life (3). Almost 30% of U.S. older adults and 37.1% (34.1–40.3%) non-institutionalized older adults in Hong Kong experienced persisting pain (4, 5). Many older adults suffer silently and needlessly, resulting in severely diminished functional ability (3), interference in daily and social activities (5), and an elevated negative feeling. Previous evidence suggested that pain conditions were associated with an elevated risk of suicidal behaviors (6–8).

Depression has been found to be associated with suicide risk in individuals with pain (9, 10). One study observed that pain–suicide association reduced when controlling for psychiatric diagnoses, including depression (7), indicating that pain might be linked to suicidality *via* mechanisms at least related to depression.

Hopelessness has been identified as relevant in the understanding of the physical pain–suicidality link (11). A large pan-Canadian study found that patients with higher pain-related helplessness and greater pain magnification were more likely to present suicidal ideation. Besides, patients with hope toward medical cure had less suicidal thoughts (9). Higher hopelessness was associated with a higher pain threshold, which correlated with less fearlessness of death (12, 13).

The interpersonal theory of suicide proposed that perceived burdensomeness is a precursor to a desire to commit suicide (14). Individuals with pain may feel that they are creating a burden on their families. One study supported the theory by showing that perceived burdensomeness significantly predicted suicidal ideation in people with chronic pain (15). Moreover, the feeling of being a burden may be exacerbated by depression and hopelessness, finally increasing the risk of carrying out a suicidal act (16).

Though the pain–suicidality link has been reported, the mechanism may be complicated. Limited studies investigated the mediation effect of psychological factors, including depression, anxiety, and sleep problem on pain and suicidality link with inconsistent results (17–19). Moreover, most studies on the pain–suicidality association focused on suicide ideation and suicide attempts.

Using a matched case–control psychological autopsy study design, the goal of the present study was to analyze the link between pain and completed suicide among Chinese rural older adults and to estimate the degree to which depressive symptoms, hopelessness, and perceived burden may explain the association.

## Materials and Methods

### Sample and Sampling

We used a multi-stage stratified cluster sampling method to select the study site. The first step was the selection of the province. We stratified 31 provinces in mainland China into three levels according to the gross domestic product per capita. Shandong province, Hunan province, and Guangxi province were chosen from the rank 1–10, 11–20, and 21–31, respectively. The second step was the selection of rural counties from the selected provinces. We stratified counties into three levels based on per capita income in each province. One county in each stratum from Shandong and Hunan and two counties in each stratum from Guangxi (as the population of the counties in Guangxi was smaller than those in the other two) were selected using a simple random sampling performed by a computer algorithm. Finally, we included 12 counties from the three selected provinces. The study was conducted from June 2014 to September 2015.

Based on the death certification system, suicide decedents aged 60 and above in rural areas (villages) were collected consecutively in each selected county. A senior suicidologist (the professor of suicidology and mental health) briefly trained all village doctors and local public health workers involved in death certification on how to determine a suicide death. They were required to report all elderly suicide deaths and possible cases that they could not determine to the local Centers for Disease Control and Prevention. After collecting all available, relevant information, trained investigators determined the manner of death.

Living comparisons were 1:1 matched with the suicides in gender, birth year (±3 years), and village. Whenever a suicide decedent was identified, the investigators would enumerate all older adults matching gender and birth year in the same village. One living comparison was then selected from the list randomly using a computer program. The investigators expanded the search to the closest village if no appropriate comparison individuals were available in the same village. Finally, we enrolled a total of 242 suicide decedents and 242 living comparisons in this study.

### Procedures of Interview

We selected two informants for each suicide decedent and living comparison. The selection of proxy informants was based on the informants' familiarity with the targets. Generally, the first informant was a next of kin who lived with the suicide decedent or the living comparison. Usually, the spouse was the first choice, followed by children and other relatives. The second informant was a friend, neighbor, or remote relative. Informants who refused to participate in the study were replaced by other suitable subjects. Among 968 informants, 450 (46.5%) were relatives of the target persons. Among 245 relatives of suicide cases, 54 (22.0%) were couples, and 95 (38.8%) sons or daughters; among 205 from controls, 80 (39.0%) were couples and 59 (28.8%) were sons or daughters. Among all informants, 232 (23.97%) informants lived together with suicides/controls before suicide/investigation [122 (23.14%) in the suicide group and 120 (24.79%) in controls].

We scheduled the interviews with the informants of suicide decedents 2–6 months after the death, while interviews with informants of living comparisons were conducted as soon as the participants and their informants were identified. Each informant was interviewed separately by one trained fieldworker, and the interviews lasted 90 min on average. The procedures of subjects' selection and interview have been detailed in a previous publication (20).

All procedures were carried out in accordance with the latest version of the Declaration of Helsinki. The study was approved by the Institutional Review Boards of the Central South University, Shandong University, and Guangxi Medical University. The investigators explained the aim and procedure of the research to all participants, and obtained written informed consent from participants in the living comparison group and all the informants of suicide decedents and living comparisons after the nature of the procedures had been fully explained.

## Measures

### Socio-Demographic Characteristics

We collected socio-demographic characteristics, including age, gender, marital status, employment status, education level, and annual family income.

### Pain Intensity

We used an 11-point visual analog scale to assess the self-perceived pain intensity. First, participants were asked, “Did you/he/she feel any pain (including any physical pain or pain with unknown reasons) before death/investigation?” Participants who endorsed “yes” were asked to report their pain intensity by circling “the one number that describes your/his/her pain on the average,” using a 0 (no pain) to 10 (the worst pain) scale (21). We categorized pain intensity into four groups: 0 means without pain, 1–3 mild pain, 4–6 moderate pain, and 7–10 severe pain. Since the number of participants in the mild pain group was small [34 (14.05%) in the suicide decedents and 31 (12.81%) in the living comparisons], we combined mild and moderate pain into a single category called “mild to moderate pain.”

### Physical Diseases

We used a question asking, “Did you/he/she have diagnosed physical diseases” to investigate whether having physical diseases before death/investigation. Physical diseases were understood as any acute or chronic illness and/or condition causing significant impairment and that may lead to long-term impairment, disability, and/or death. Investigators recorded diagnoses of physical diseases made by physicians, and reviewed medical records with consent when necessary. Zero means without physical disease, 1 means with one or more physical diseases.

### Social Support

We used the 23-item Duke Social Support Index (DDSI) to measure social support in the last week before suicide/investigation. A total score ranges from 11 to 45, with a higher score indicating higher social support. DSSI has been used in a previous psychological autopsy study in China and showed satisfactory reliability and validity (22). We dichotomized social support into two groups based on the median score of DSSI.

### Perceived Burden

We used the question that “Did you/he/she feel that caring you/he/she bring mental or physical burden to your/his/her family?” to rank the perceived burden level. One means “no perceived burden,” and 4 means “heavy perceived burden.” Perceived burden was categorized into two groups (1 means without perceived burden; 2–4 means with perceived burden).

### Depressive Symptoms

We used the Geriatric Depression Scale (GDS-30) to assess the depressive symptoms in the last week before death/investigation (23). A higher score means more severe depressive symptoms. A cutoff score of 10 and 20 represents mild and severe levels of depressive symptoms, respectively. The GDS-30 has shown adequate properties in the Chinese elderly (23). In the conditional logistic regression, GDS score was categorized into two groups: 0–10 means without depressive symptoms; 11–30 means with depressive symptoms.

### Hopelessness

We used the Beck's Hopelessness Scale (BHS-4) to measure hopelessness in the last week before death/investigation (24). It consists of four items relevant to success, dark future, breaks, and faith. Each item is rated on a five-point scale ranging from 1 (strongly agree) to 5 (strongly disagree). A total score ranges from 4 to 20, with a higher score representing a higher level of hopelessness (24). We categorized the severity of hopelessness into two groups according to the median of BHS score.

### Data Combination

We combined the information provided by the two informants as the proxy data for each suicide decedent and living comparison. For sociodemographic characteristics, we relied on the data provided by the first informant. Regarding psychosocial factors, we used information that was hypothetically associated with elevated suicide risk, considering any targeting symptoms or behaviors might exist as long as any informant may have observed (20, 25). Hence, we used the higher score of pain intensity, perceived burden, and BHS-4, and the lower score of DSSI for analysis; similarly, a positive answer of an item of GDS-30 was used if one of the two informants reported positive. We used the proxy data for analysis.

Although we could not confirm whether the suicide decedents actually had those symptoms, such as depressive symptoms, all analyses were conducted under the premise that the validity of proxy-based information has been proved fair. We used the intraclass correlation coefficient (ICC) to evaluate the agreement between subject-based and proxy-based information. For the GDS-30, subject–proxy concordance was fair (ICC = 0.590). Based on the proxy data, Cronbach's alpha was 0.871 among suicide decedents and 0.889 among the living comparisons, demonstrating good internal reliability (26). For BHS-4, the subject–proxy agreement was good (ICC = 0.682). The Cronbach's alpha was 0.834 (27). Moreover, the subject–proxy agreements on pain intensity (ICC = 0.504) and perceived burden (ICC = 0.487) were fair. Nonetheless, the validity of proxy evaluation should be considered with extreme caution.

### Statistical Analyses

Descriptive analysis, chi-squared tests for categorical variables, and Student's *t*-tests for continuous variables were used to describe and compare the socio-demographics, pain intensity, perceived burden, hopelessness, and depressive symptoms of suicide cases and living controls. Conditional logistic regression (backward stepwise selection) was used to assess the association between pain intensity and completed suicide. Covariates adjusted were as follows: marital status, employment status, education level, annual family income, physical disease, social support, depressive symptoms, hopelessness, and perceived burden.

Mediation analysis was conducted to assess the specific contribution of depressive symptoms, hopelessness, and perceived burden in the pain–suicide association. The KHB method can be used to decomposes total effect (the effect of pain on suicide without adjustment of mediators) into direct effect (the effect of pain on suicide) and indirect effects (the mediation effects from depressive symptoms, hopelessness, and perceived burden) in conditional logistic regression (28, 29). Moreover, the confounding percentage to total effect due to mediators and the disentangle contribution of each mediator can be calculated. Marital status, employment status, education level, annual family income, physical disease, and social support were controlled in the mediation analysis. The mediation analysis was performed with the khb command. Entering method, rather than backward stepwise selection, was used in mediation analysis since the khb command is not supported by stepwise method. All analysis was performed using Stata 16.0 (College Station, Texas 77845, USA). The level of statistical significance was set at *p* < 0.05.

## Results

### Characters of Suicide Decedents and Living Comparisons

We investigated 242 suicide decedents and 242 living decedents. The mean age was 74.4 ± 8.2 years, and 55.8% were male among the suicides. The mean age was 74.1 ± 8.2 years, and 55.8% were male among living comparisons. Compared with the living controls, the suicides tended to be not currently married, unemployed, and with physical disease (all *p*-values <0.05). Suicide cases reported higher pain intensity, more perceived burden, higher level of depressive symptoms and hopelessness, and lower level of social support than living controls (all *p*-values <0.01). Descriptive data for study variables are presented in Table 1.

**Table 1:** Characters of suicide cases and living controls.

**Variables**	**Suicide cases (*n* = 242)**	**Living controls (*n* = 242)**	***z*/*t*/χ^2^**	***p***
Age [mean (S.D.)]	74.4 (8.2)	74.1 (8.2)	0.51	0.609
Gender				
Male	135 (55.8)	135 (55.8)		
Female	107 (44.2)	107 (44.2)		
Marital status			19.89	<0.001
Currently married	122 (50.4)	170 (70.2)		
Not currently married	120 (49.6)	72 (29.8)		
Employment status			8.27	0.016
Employed	41 (16.9)	61 (25.2)		
Unemployed	194 (80.2)	167 (69.0)		
Retired	7 (2.9)	14 (5.8)		
Education			1.92	0.383
Blow primary school	111 (45.9)	96 (39.7)		
Primary school or above	105 (43.4)	116 (47.9)		
Above primary school	26 (10.7)	30 (12.4)		
Physical disease			18.52	<0.001
Without	40 (16.53)	81 (33.47)		
Any	202 (83.47)	161(66.53)		
Social support [mean (S.D.)]	22.9 (6.0)	27.5 (6.8)	7.87	<0.001
Lower group ( ≤ 25)	166 (68.60)	95 (39.26)	41.92	<0.001
Higher group (>25)	76 (31.40)	147 (60.74)		
Pain intensity [mean (S.D.)]	4.0 (3.8)	2.5 (3.0)	4.85	<0.001
None (0)	93 (38.43)	124 (51.24)	45.18	<0.001
Mild to moderate (1–6)	59 (24.38)	91 (37.60)		
Severe (7–10)	90 (37.19)	27 (11.16)		
Perceived burden [mean (S.D.)]	2.1 (1.3)	1.3 (1.1)	7.43	<0.001
Without (=1)	72 (29.75)	130 (53.72)	28.58	<0.001
With (>1)	170 (70.25)	112 (46.28)		
Depressive symptom [mean (S.D.)]	21.4 (5.9)	9.2 (6.4)	21.65	<0.001
Without ( ≤ 10)	15 (6.20)	156 (64.46)	179.78	<0.001
With (>10)	227 (93.80)	86 (35.54)		
Hopelessness [mean (S.D.)]	14.6 (2.5)	9.7 (2.7)	20.42	<0.001
Lower group ( ≤ 12)	50 (20.66)	207 (85.54)	204.49	<0.001
Higher group (>12)	192 (79.34)	35 (14.46)		

### Logistic Regression Analysis

Conditional logistic regression was employed to detect risk factors of suicide completion among older adults in rural China. As shown in Table 2, unemployment [odds ratio (OR) = 5.06, 95% confidence interval (CI): 1.76–14.49], higher level of hopelessness (OR = 7.72, 95% CI: 3.49–17.10), depressive symptom (OR = 15.82, 95% CI: 4.53–55.25), and severe pain (OR = 3.46, 95% CI: 1.31–9.13) were significantly associated with suicide completion. Mild to moderate (OR = 0.54; 95% CI: 0.20–1.43) pain was not significantly associated with suicide completion.

**Table 2:** Multivariate logistic regression for completed suicide among the rural elderly in China.

**Independent variables**	**OR**	**95% CI**	***p***
Employment status			
Employed	1.00 (ref)	-	-
Unemployed	5.06	1.76–14.49	0.003
Retired	2.13	0.25–18.09	0.490
Hopelessness			
Lower group ( ≤ 12)	1.00 (ref)	–	–
Higher group (>12)	7.72	3.49–17.10	<0.001
Depressive symptoms			
Without ( ≤ 10)	1.00 (ref)	–	–
With (>10)	15.82	4.53–55.25	<0.001
Pain intensity			
None (0)	1.00 (ref)	–	–
Mild to moderate (1–6)	0.54	0.20–1.43	0.213
Severe (7–10)	3.46	1.31–9.13	0.012

*Adjusted for education level, employment, marital status, annual family income, physical disease, social support, perceived burden, hopelessness, and depressive symptoms. OR, odds ratio; CI, confidence interval. R^2^ = 0.7084*.

### Mediation Analysis

The results of the mediation analysis are shown in Table 3. To simplify interpretation of OR, pain intensity was dichotomized as “moderate or less” and “severe” because increased risk of suicide completion was observed only in the older adults with severe pain. Depressive symptoms, hopelessness, and perceived burden significantly mediated the association between pain and completed suicide (*p* = 0.020). The total effect of pain intensity on suicide completion was 1.78 times larger than the direct effect, and 43.71% of the total effect was due to mediators (indirect effect). In terms of the specific contributions of mediators, depressive symptoms explained 17.39% of the association between severe pain and suicide completion, hopelessness explained 17.63%, and perceived burden explained 8.69%. Depressive symptoms alone could not significantly mediate pain–suicide association. Nor could hopelessness, or perceived burden. Any combinations of two factors did not have a significant mediational effect (all *p* > 0.05) (Supplementary Material).

**Table 3:** Mediation analysis for the association between pain and completed suicide.

**Effects**	**OR**	**95% CI**	***p***
Total effect	14.46	4.51–46.32	<0.001
Direct effect	4.50	1.47–13.73	0.008
Indirect effect	3.21	1.21–8.56	0.020

*Exposure: pain intensity, outcome: completed suicide, mediators: depressive symptoms, hopelessness, and perceived burden. Covariates: education level, employment, marital status, annual family income, physical disease, and social support. The total effect is 1.78 times larger than the direct effect, and 43.71% of the total effect is due to mediators, with 17.39% due to depressive symptoms, 17.63% due to hopelessness, and 8.69% due to perceived burden*.

## Discussion

As of our knowledge, our study is the first one to use suicide completion as the outcome to investigate the mediation effect of depressive symptoms, hopelessness, and perceived burden on the pain–suicide association and to quantify the individual mediational effect. We find that pain was an independent risk factor of suicide completion in older adults in rural China. Furthermore, our results indicated that depressive symptoms, hopelessness, and perceived burden significantly but partially mediated the pain–suicide association.

Previous studies found that patients with pain had increased risk of suicidal outcomes compared with the general population. A large cohort study demonstrated that veterans with self-reported severe pain were more likely to die by suicide than patients experiencing moderate or less pain (HR: 1.33, 95% CI: 1.15–1.54) (30). A case–control study among older adults in Canada also found that severe pain (OR: 4.07, 95% CI: 2.51–6.59) elevated the risk of suicide death (31). However, the study only considered patients with prescribed pain medication. Consistent with previous studies, the present study found that severe pain was more common in suicide cases than in controls (37.19% vs. 11.16%). We proved that older adults with severe pain predicted 3.46 times of suicide completion risk than those without pain. Older adults with severe pain may view suicide as a way to escape from suffering (32). The risk for suicide among the older population with severe pain merits particular attention.

One major finding was that 56.29% of the total effect of pain intensity on suicide was from pain directly, and perceived burden, hopelessness, and depressive symptoms significantly mediated 43.71% of the association between pain and suicide. These results were consistent with one recent study proving that depression and anxiety disorders had mediational effects on the relationships between pain and suicidal behaviors (17). Though this study only used suicide ideation and suicide attempt as outcomes, the study used a large, representative sample of the English adult population to prove the partial mediation effect of depression. However, several studies found different results that the pain–suicide ideation relationship was fully mediated by depression (18, 33). A possible reason for this discrepancy might be due to the different outcomes (suicide completion vs. suicidal ideation/attempt) and different populations across these studies.

Moreover, we measured the individual mediational effect. Depressive symptoms, hopelessness, and perceived burden explained 17.39, 17.63, and 8.69% of the total effect of pain intensity on suicide completion, respectively. Many studies suggested that hopelessness significantly predicted suicidal behaviors. A meta-analysis of longitudinal studies found that hopelessness significantly predicted suicidal ideation, suicide attempt, and suicide death (34). Moreover, helplessness/hopelessness in patients with pain has been reported as a significant predictor of suicidal ideation in previous studies (9, 15, 35). Our results suggest that further investigation should focus more on hopelessness in the pain–suicide association.

Consistent with prior evidences (36), we also found the mediating effect of the perceived burden on the pain–suicide association. The interpersonal theory of suicide proposed that thwarted belongingness and perceived burdensomeness were key factors contributing to a suicide desire (14). Perceived burden has been identified as a critical predictor of suicidality in patients with pain (15, 37). It is reasonable to expect that the older adults suffering from pain felt that they created a burden to their family. The current finding provided further evidence for the need to consider perceived burdens in preventing suicide for older adults with pain in rural areas.

Previous studies have reported depression as the most common mental illness among suicide decedents (20, 38, 39). Moreover, higher pain severity was a significant predictor of the onset of depression (40). Our results are in line with previous evidence. We further demonstrated that depression partially mediated the impact of pain on suicide.

Interestingly, we found that only depressive symptoms could not mediate the association between pain and suicide completion. Nor did the perceived burden or hopelessness. The relationship between pain and suicide is complex and involves several psychological and psychosocial factors (32). Moreover, those factors might interact with each other, and the interactions may affect the pain–suicidality relationship. A study in back pain patients found that pain intensity showed indirect effects due to pain-related helplessness/hopelessness on depression (41). Depressive symptoms were found to be a significant predictor of self-perceived burdensomeness in patients with chronic pain (42). Furthermore, a community-based study suggested that hopelessness and the interaction between perceived burdens and hopelessness were significantly associated with suicide plans and attempts (43).

Depression, hopelessness, and perceived burden might interact with and exacerbate each other, finally increasing the vulnerability to commit suicide. Though we found the mediators and their respective indirect effects, further studies on the interactions among mediators are needed for a comprehensive understanding of pain–suicide association.

The current study has several limitations. First, though psychological autopsy is a widely used method to explore the risk factors of completed suicide, the validity of data provided by proxy informants is a concern. However, previous studies had proved that subject–proxy concordance for the GDS-30 and BHS-4 were fair in the living controls (26). Second, we measured pain with a single question concerning the intensity of pain without data on the location, type, and duration. More detailed questions on pain might be better for the comprehensive understanding between pain and suicide completion. However, most research generally examines only a limited number of types or locations of pain. Moreover, a review had suggested that pain itself, irrespective of which type or location, was a determinant of suicidality based on abundant evidence (44). Furthermore, it seems that there is no strong link between pain duration and suicidality. As the duration of suffering pain increases, suicidal behaviors may be less related to pain symptoms (44). Also, prior studies had demonstrated that sleep disturbance was a mediator in the pain–suicidality link (19). Nevertheless, we did not include information on sleep quality. Further research is warranted to examine this possibility. Finally, we estimated the pain–suicide death association and mediational effects in older adults in rural China. The results might not be suitable to generalize to older adults in other areas and should be interpreted with caution.

## Conclusions

Despite these limitations, our study added to a growing body of evidence indicating that pain independently increased the risk of committing suicide and that perceived burden, hopelessness, and depressive symptoms partially mediated the pain–suicide association. Given the undertreated pain in late life and backward mental health services in rural areas, regular screening of pain, depressive symptoms, hopelessness, and perceived burden using simple but sensitive questions or scales for older adults with pain is vital for the prevention and early detection of suicide risk. Furthermore, treatment of pain and psychological interventions for alleviating depressive symptoms and feelings of hopelessness are critical for suicide prevention.

## Data Availability Statement

The original contributions presented in the study are included in the article/Supplementary Material, further inquiries can be directed to the corresponding author.

## Ethics Statement

The studies involving human participants were reviewed and approved by Institutional Review Boards of Central South University, Shandong University, and Guangxi Medical University. The patients/participants provided their written informed consent to participate in this study.

## Author Contributions

CJ, ZM, and LZ contributed to the study design. GW contributed to data collection. JW and JX conducted the data analysis. JW drafted the article. LZ provided substantial editorial input in the drafting of the manuscript. All authors read and approved the final manuscript.

## Funding

This work was supported by the American Foundation for Suicide Prevention [Grant No. SRG-0-169-12] and Science and Technology Plan Project of Guangdong Province [No. 2019B030316001].

## Conflict of Interest

The authors declare that the research was conducted in the absence of any commercial or financial relationships that could be construed as a potential conflict of interest.

## Publisher's Note

All claims expressed in this article are solely those of the authors and do not necessarily represent those of their affiliated organizations, or those of the publisher, the editors and the reviewers. Any product that may be evaluated in this article, or claim that may be made by its manufacturer, is not guaranteed or endorsed by the publisher.
