# Supplementary material for: The Mediating Role of Depressive Symptoms, Hopelessness, and Perceived Burden on the Association Between Pain Intensity and Late-Life Suicide in Rural China: A Case–Control Psychological Autopsy Study
Source: Front Psychiatry. 2021 Dec 13;12:779178. doi: 10.3389/fpsyt.2021.779178 (PMC8710711; doi:10.3389/fpsyt.2021.779178)
Supplement: Supplementary file 1 [file Table_1.DOCX]

Supplementary materials

**Table 1 Mediation analysis for the association between pain and completed suicide.**

| Effects | OR | 95%CI | *P* |
| --- | --- | --- | --- |
| Total effect | 5.06 | 1.64- 15.57 | 0.005 |
| Direct effect | 4.50 | 1.47- 13.73 | 0.008 |
| Indirect effect | 1.12 | 0.66- 1.91 | 0.664 |

Exposure: pain intensity, outcome: completed suicide, mediator: depressive symptoms. Covariates: education level, employment, marital status, annual family income, physical disease, social support, hopelessness, and perceived burden.

**Table 2 Mediation analysis for the association between pain and completed suicide.**

| Effects | OR | 95%CI | *P* |
| --- | --- | --- | --- |
| Total effect | 5.23 | 1.70- 16.10 | 0.004 |
| Direct effect | 4.50 | 1.47- 13.73 | 0.008 |
| Indirect effect | 1.16 | 0.71- 1.89 | 0.546 |

Exposure: pain intensity, outcome: completed suicide, mediator: hopelessness. Covariates: education level, employment, marital status, annual family income, physical disease, social support, depressive symptoms, and perceived burden.

**Table 3 Mediation analysis for the association between pain and completed suicide.**

| Effects | OR | 95%CI | *P* |
| --- | --- | --- | --- |
| Total effect | 5.50 | 1.90- 15.94 | 0.002 |
| Direct effect | 4.50 | 1.47- 13.73 | 0.008 |
| Indirect effect | 1.223 | 0.87- 1.72 | 0.245 |

Exposure: pain intensity, outcome: completed suicide, mediator: perceived burden. Covariates: education level, employment, marital status, annual family income, physical disease, social support, depressive symptoms, and hopelessness.

**Table 4 Mediation analysis for the association between pain and completed suicide.**

| Effects | OR | 95%CI | *P* |
| --- | --- | --- | --- |
| Total effect | 7.47 | 2.35- 23.72 | 0.001 |
| Direct effect | 4.50 | 1.47- 13.73 | 0.008 |
| Indirect effect | 1.66 | 0.66- 4.16 | 0.280 |

Exposure: pain intensity, outcome: completed suicide, mediators: depressive symptoms, hopelessness. Covariates: education level, employment, marital status, annual family income, physical disease, social support, and perceived burden.

**Table 5 Mediation analysis for the association between pain and completed suicide.**

| Effects | OR | 95%CI | *P* |
| --- | --- | --- | --- |
| Total effect | 6.84 | 2.32- 20.19 | 0.001 |
| Direct effect | 4.50 | 1.47- 13.73 | 0.008 |
| Indirect effect | 1.52 | 0.83- 2.77 | 0.172 |

Exposure: pain intensity, outcome: completed suicide, mediators: depressive symptoms, perceived burden. Covariates: education level, employment, marital status, annual family income, physical disease, social support, and hopelessness.

**Table 6 Mediation analysis for the association between pain and completed suicide.**

| Effects | OR | 95%CI | *P* |
| --- | --- | --- | --- |
| Total effect | 7.21 | 2.43- 21.39 | 0.001 |
| Direct effect | 4.50 | 1.47- 13.73 | 0.008 |
| Indirect effect | 1.60 | 0.89- 2.90 | 0.118 |

Exposure: pain intensity, outcome: completed suicide, mediators: perceived burden, hopelessness. Covariates: education level, employment, marital status, annual family income, physical disease, social support, and depressive symptoms.
